# Value of Speckle Tracking Echocardiography Combined with Stress Echocardiography in Predicting Surgical Outcome of Severe Aortic Regurgitation with Markedly Reduced Left Ventricular Function

**DOI:** 10.31083/j.rcm2404114

**Published:** 2023-04-17

**Authors:** Quan Li, Wuxu Zuo, Yu Liu, Beiqi Chen, Yuanfeng Wu, Lili Dong, Xianhong Shu

**Affiliations:** ^1^Department of Echocardiography, Zhongshan Hospital, Fudan University, 200032 Shanghai, China; ^2^Shanghai Institute of Cardiovascular Diseases, 200032 Shanghai, China; ^3^Shanghai Institute of Medical Imaging, 200032 Shanghai, China

**Keywords:** speckle tracking echocardiography, stress echocardiography, aortic regurgitation, left ventricular systolic function, LVEF, longitudinal strain

## Abstract

**Background:**

Predicting outcomes of surgical aortic valve replacement (AVR) in patients with chronic severe aortic regurgitation (AR) and markedly reduced left ventricular (LV) function remains a challenge. This study aimed to explore the preoperative echocardiographic index that could predict the recovery of LV systolic function after surgery in patients with chronic severe AR and reduced left ventricular ejection fraction (LVEF).

**Methods:**

The study group consisted of 50 patients diagnosed with chronic severe AR (>6 months) and significantly reduced LVEF (18~35%, average 26.2 ± 5.3%). Low-dose dobutamine stress echocardiography (DSE) was performed before surgery. Only patients with an absolute increase in LVEF ≥8% during DSE were referred for surgical AVR. During following up (over six months to one year after surgery), the patients were divided into two groups by postoperative LVEF (> or ≤40%). DSE- and speckle tracking echocardiography (STE)-derived LV functional parameters were compared between groups to identify predictors of post-operative improvement in LVEF.

**Results:**

A total of 38 patients underwent AVR. One patient died before discharge. Post-surgical LV size and LVEF improved markedly after surgery in all patients (n = 37). Pre-surgical LV end-systolic diameter, baseline global longitudinal strain (GLS) and peak GLS were better in the group with LVEF >40% (n = 18; *p *< 0.05, *t* test). Baseline GLS and peak GLS correlated moderately with post-surgery LVEF (R = –0.581, *p *< 0.001; R = –0.596, *p *< 0.001; respectively). Logistic regression analysis demonstrated baseline GLS and peak GLS were the independent predictors of post-surgery improvement of LVEF. Peak GLS had the highest prediction value (area under the curve = 0.895, sensitivity and specificity: 89.5% and 77.8%, respectively), with a cutoff value of –9.4%.

**Conclusions:**

This study shows that STE combined with DSE can provide sensitive quantitative indices for predicting improvement of LV systolic function after AVR in patients with chronic severe AR and significantly decreased LVEF.

## 1. Introduction

Chronic severe aortic regurgitation (AR) has a poor prognosis and is associated with increased mortality and morbidity [1]. At its late stage, the markedly decreased left ventricular ejection fraction (LVEF) may incur excessive surgical mortality. However, previous studies found that surgical aortic valve replacement (AVR) could still be beneficial since volume overload is relieved [2, 3, 4]. Therefore, it is of great importance to identify those preoperative parameters which distinguish those patients that can have a better recovery, which is closely related to improvement of symptoms and long-term prognosis [5, 6, 7].

Stress echocardiography (SE) has been used to identify viable myocardium and contractile reserve (CR) in a variety of heart diseases with left ventricular (LV) contractile dysfunction [8, 9, 10]. In previous studies, LV CR estimated by low-dose dobutamine stress echocardiography (DSE) in patients with severe AR and mild-moderately reduced LVEF is highly predictive of postoperative LV contractile function and clinical outcomes after AVR [11, 12]. In patients with chronic severe AR with significantly decreased LVEF, however, it remains unclear whether low-dose DSE has the same predictive power in the recovery of LV contractile function after AVR.

Speckle tracking echocardiographic (STE) is a reliable and reproducible method to assess myocardial deformation with incremental value to subtle regional wall motion change than traditional echocardiography, and it had been shown to achieve high reproducibility during all stages of SE [13, 14, 15]. In the present study, by combining STE and low-dose DSE, we sought to determine novel predictors for early recovery of LV contractile function following surgical AVR in patients with chronic severe AR and significantly decreased LVEF.

## 2. Methods

From April 2014 to February 2018, 50 patients with chronic severe AR and a significant reduction of LVEF (<35%) were recruited from Zhongshan Hospital, Fudan university. All patients underwent outpatient conventional transthoracic echocardiography (TTE) to identify candidates for enrollment. The severity of AR was determined combining the qualitative, quantitative and semiquantitative indices from conventional TTE: vena contracta width, pressure half-time, effective regurgitant orifice area, regurgitant volume, LV dimension, and holodiastolic flow reversal in the descending aorta [16].

Exclusion criteria included concomitant moderate or severe aortic stenosis and other moderate or severe valvular disease, coronary artery disease, atrial fibrillation, congenital heart disease, cardiomyopathy, severe hypertension, allergies to dobutamine, and other systemic diseases that cannot tolerate DSE. This study was conducted according to the principles stated in the Declaration of Helsinki. Ethics approval for the study was granted by the Ethics Committees of Zhongshan Hospital, Fudan University, and all patients provided written informed consent.

### 2.1 Dobutamine Stress Echocardiography

All patients underwent low-dose dobutamine stress echocardiography** (**DSE) (peak dose 20 μg/kg/min) using a standard protocol with an incremental dobutamine infusion rate of 5, 10, 20 μg/kg/min every 5 minutes [10]. Criteria for terminating the test were typical angina symptoms, any refractory symptoms (such as headache, nausea, and vomiting), obvious arrhythmia (frequent ventricular premature beats, ventricular velocity), severe hypertension (systolic blood pressure >220 mmHg or diastolic blood pressure >110 mmHg), or blood pressure reduction (20 mmHg lower than before the study).

**Fig. 1.**
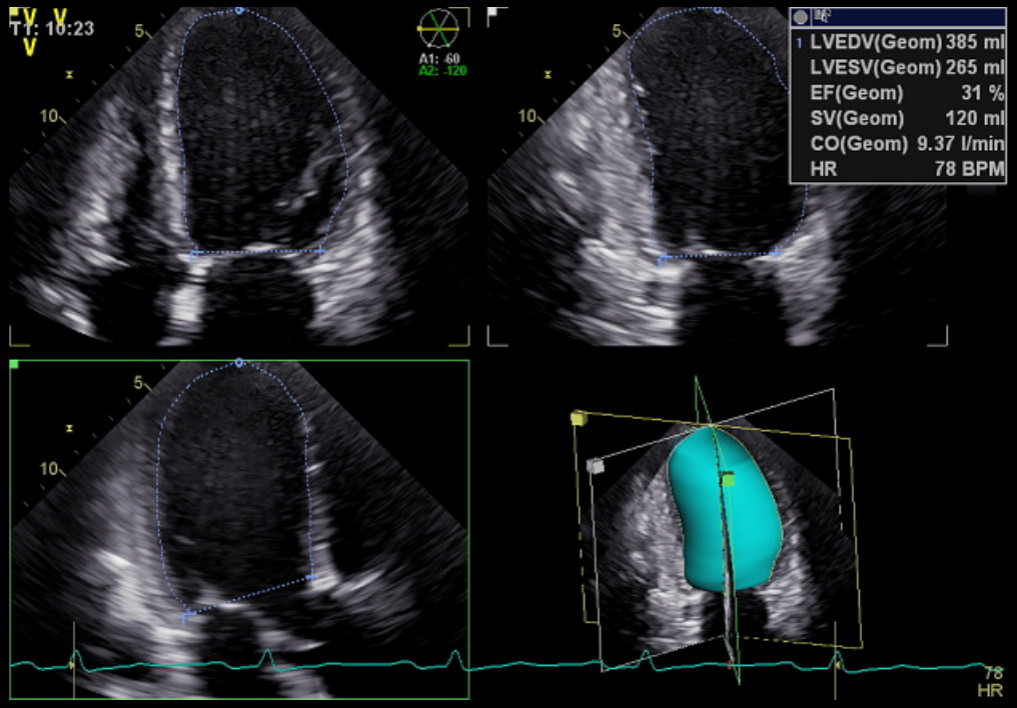
**Representative example of LV volume analyzed through triplane Simpson method, with subsequent calculation of LVEF**. LV, left ventricular; LVEF, left ventricular ejection fraction.

Examinations were performed with a GE E9 system equipped with a M5Sc probe (1.7–3.4 Hz) (GE Vingmed Ultrasound AS, Horten, Norway). Image acquisition and conventional measurements were performed according to the American Society of Echocardiography Examination guidelines for adult transthoracic echocardiography [17]. Dynamic images were acquired in cine loops with 3–5 cardiac cycles for on-cart analysis during rest and peak stress stage. All echocardiographic images were recorded in a digital raw-data format (native DICOM format) for further analysis. During the comprehensive echocardiographic examination, LV end-diastolic diameter (LVEDD) and LV end-systolic diameter (LVESD) were obtained by M-mode in parasternal long axis view at rest (defined as baseline or pre-surgery values, respectively). Standard two-dimensional (2D) apical views (four-chamber, two-chamber, and three-chamber) were obtained in the triplane mode using a three-dimensional (3D) matrix array transducer. LV end-diastolic volume, LV end-systolic volume and stroke volume were analyzed using the triplane Simpson method, with subsequent calculation of LVEF at rest and peak stress stage (defined as baseline or pre-surgery LVEF value, peak LVEF value, respectively) (Fig. 1). The above indicators are the average of 3 consecutive cardiac cycles. Current guidelines recommend the LV CR definition in asymptomatic chronic AR patients as an absolute increase in ejection fraction (ΔLVEF) ≥5% [10]. However, the current study population were highly symptomatic and thus at a later stage in the disease progress and recommendation for such patients was still lacking. The adoption of a cut-off of 5% would expose the patients to unnecessary surgical risks. So, a definition of ΔLVEF ≥7.5% was considered as it was the strictest cut-off for inadequate LV CR recommended by guidelines. At our center, an adequate LV CR was defined as ΔLVEF ≥8% in the current study for easier clinical application.

### 2.2 Speckle Tracking Echocardiography

**Fig. 2.**
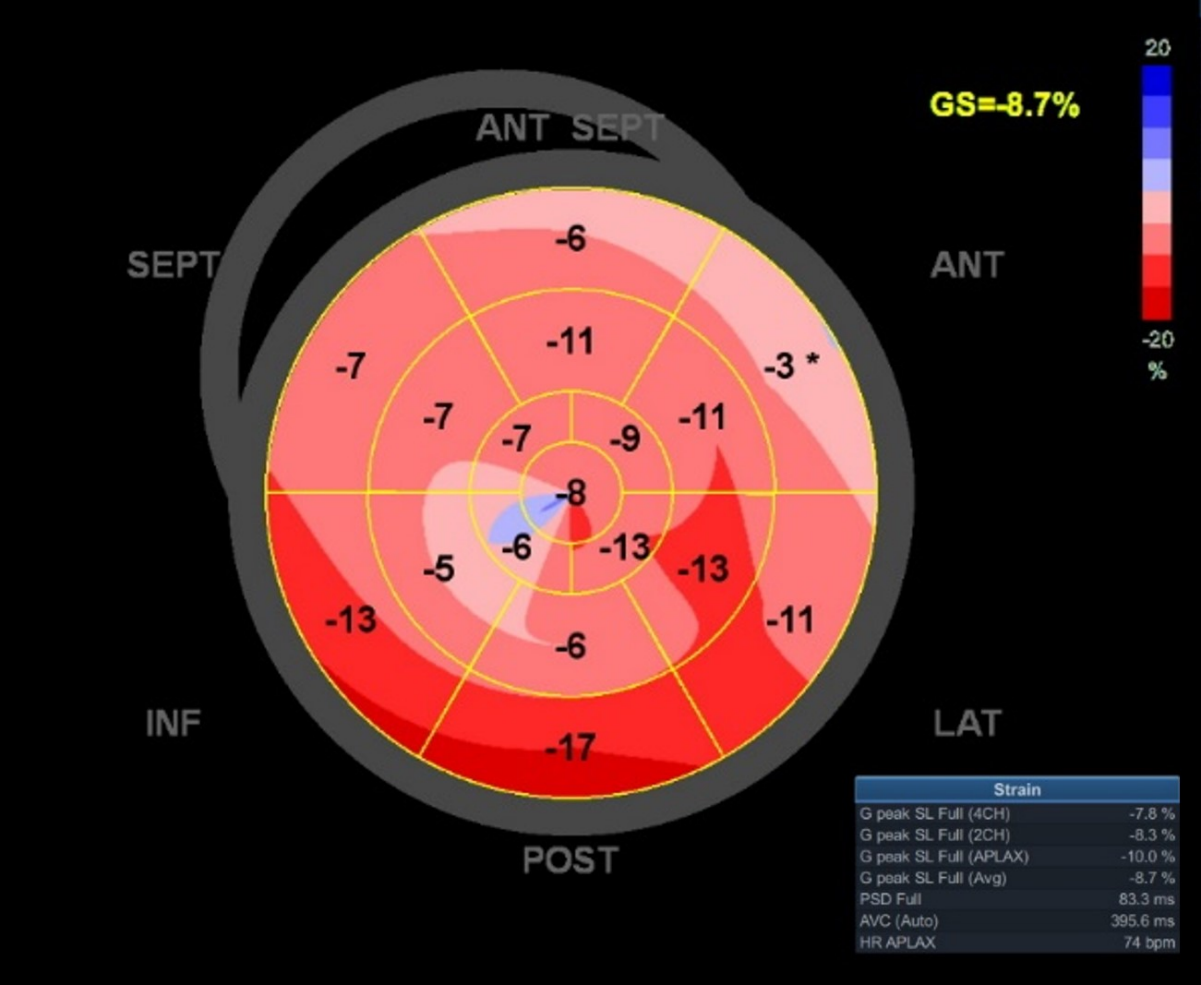
**Representative example of the LV GLS measurement based on 2D echocardiography by offline analysis software EchoPAC**. LV, left ventricular; GLS, global longitudinal strain; 2D, two-dimensional.

The analysis was performed offline by a single observer without knowledge of hemodynamic data, using commercially available software (Echopac PC, Version 203, GE Vingmed Ultrasound AS, Horten, Norway). The LV global longitudinal strain (GLS) was analyzed in 2D images of three apical views (four-chamber, two-chamber, and three-chamber) at rest and peak stress stage during low-dose DSE (defined as baseline GLS and peak GLS values, respectively). The software could track the motion of speckles within the myocardium after the LV endocardial border was delineated in the end-systolic frame, and automatically analyze the longitudinal strain. If the tracking is suboptimal, the region of interest can be readjusted in real-time. After obtaining the corresponding curves and longitudinal strain values of the three apical views, the software could automatically calculate the LV GLS, which was the consecutive average of the peak systolic longitudinal strain (Fig. 2).

### 2.3 Aortic Valve Replacement and Follow-Up

All the patients with LV CR underwent standard surgical AVR. Perioperative events that were recorded included death, infection, heart failure, prolonged ventilation, and other cardiovascular and cerebrovascular events. Follow-up TTE was performed over six months to one year, and included LVEF, LVEDD and LVESD (defined as post-surgery values, respectively).

**Fig. 3.**
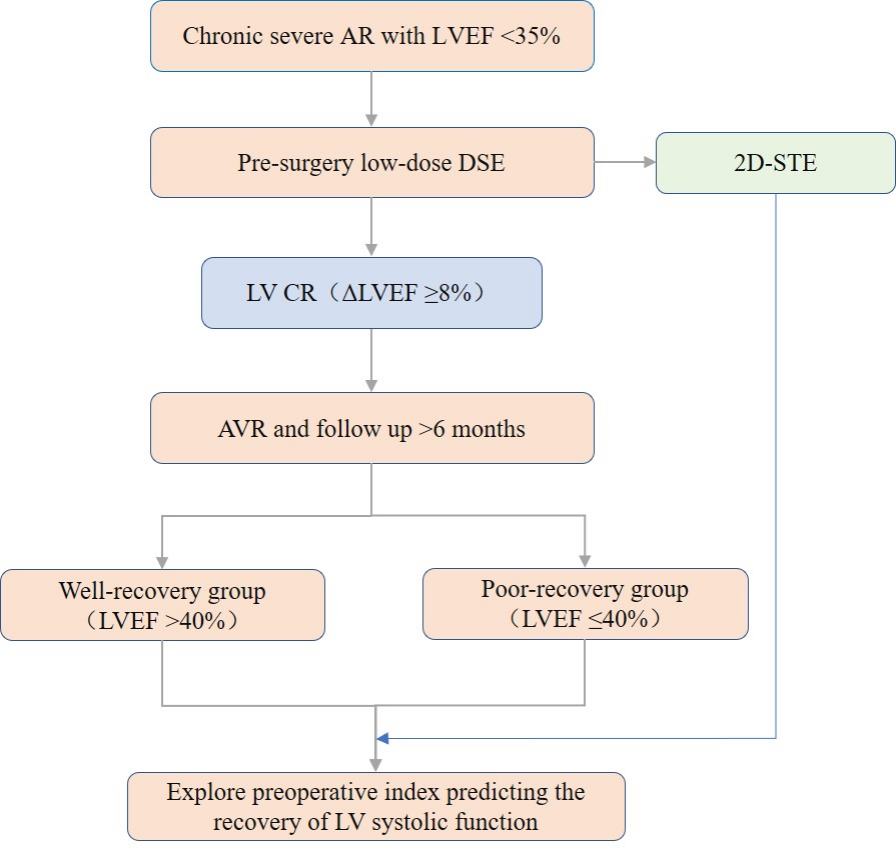
**The experimental flow chart**. AR, aortic regurgitation; LVEF, left ventricular ejection fraction; DSE, Dobutamine stress echocardiography; 2D-STE, two-dimensional speckle tracking echocardiographic; LV, left ventricular; CR, contraction reserve; AVR, aortic valve replacement.

During the follow-up period, patients were divided into two groups according to whether the post-surgery LVEF improved to the lower limit of heart failure with mildly reduced LVEF (HFmrEF, which is defined as LVEF 41%–49% according to the 2022 AHA/ACC/HFSA guideline) [18]. The well-recovery group was defined as an LVEF >40% and the poor-recovery group was defined as LVEF ≤40%. The experimental flow chart is shown in Fig. 3.

### 2.4 Statistical Analysis

Statistical analysis was performed using SPSS version 22.0 (SPSS, Chicago, IL, USA). All continuity variables were tested by normality test and presented as the mean ± standard deviation (SD). The paired *t* test was used to compare the pre-surgery and post-surgery measurements. Differences between the two groups were analyzed using independent samples *t* test. Chi-square test was performed for categorical variables. The Mann-Whitney U test was used for non-normally distributed continuous variables. Simple linear regression analysis was used to determine correlation between pre-surgery variables and post-surgery LVEF. A logistic regression model analysis was performed to identify independent correlates of the post-surgery LVEF. Analysis of the receiver-operating characteristic (ROC) curve was used to assess the ability of pre-surgery parameters for predicting the well-recovery patients. The cutoff value was calculated by determining the pre-surgery parameters that provided the greatest reference value of sensitivity and specificity. For all statistical comparisons, *p *< 0.05 was considered as significant differences.

## 3. Results

### 3.1 Study Population

A total of 50 patients were included in this retrospective study. All patients achieved the peak stress (20 μg/Kg/min) during low-dose DSE without any complications. Among these patients, 12 patients without LV CR did not undergo surgical AVR. 38 patients underwent standard surgical AVR; one patient died (2.7%) because of respiratory failure during the perioperative period. 37 patients (baseline LVEF 18~35%, average 26.2 ± 5.3%; 87% male) successfully underwent AVR (76% mechanical valve and 24% biological valve). All patients received guideline-directed anti-heart failure therapy after AVR. A standard regime at our center during the study period included beta-blocker, angiotensin converting enzyme inhibitor, and diuretics and the dosage would be titrated as per patient tolerance. A total of 4 patients (10.8%) developed atrial fibrillation during the follow-up period. The basic clinical data, type of lesion, and procedural characteristics of the study population (n = 37) are shown in Table 1. The patients were followed up by TTE over a mean period of 8.7 ± 2.8 months (median 8 months).

**Table 1.** ** Baseline characteristics of the study population**.

Vriables		Patients (n = 37)
Age (yrs)		55 ± 10
Male, n (%)		32 (87)
Hypertension, n (%)		14 (38)
Diabetes, n (%)		5 (14)
NYHA class (≥II), n (%)		30 (81)
Heart rate (bpm)		76 ± 12
Etiology		
	Degenerative, n (%)	13 (35)
	Bicuspid valve, n (%)	6 (16)
	Endocarditis, n (%)	1 (3)
	Rheumatic, n (%)	6 (16)
	Aortic root ectasia, n (%)	11 (30)
Surgical method		
	AVR, n (%)	29 (74)
	Bentall, n (%)	8 (26)
Valve type		
	Mechanical valve, n (%)	28 (76)
	Biological valve, n (%)	9 (24)

Data are expressed as mean ± SD or as n (%). NYHA, New York Heart Association; AVR, aortic valve replacement.

### 3.2 Characteristics during Low-Dose DSE

Table 2 shows the changes of LVEF and GLS at rest and peak stress stage during DSE. Baseline LVEF and baseline GLS were all significantly lower than normal levels. The peak values during DSE significantly increased compared with those at rest (36.1 ± 6.1 *vs.* 26.2 ± 5.3 for LVEF, *p *< 0.001; –9.4 ± 1.8 *vs.* –7.5 ± 1.8 for GLS, *p *< 0.001, respectively), but still significantly lower than normal levels. The average ΔLVEF and ΔGLS were 10.0% and –1.9%, respectively.

**Table 2.** **Changes of LVEF and GLS during DSE (n = 37)**.

Variables	Baseline	Peak	Addition (Δ)
LVEF (%)	26.2 ± 5.3	36.1 ± 6.1*	10.0 ± 2.8
GLS (%)	–7.5 ± 1.8	–9.4 ± 1.8*	–1.9 ± 1.0

Data are expressed as mean ± SD. LVEF, left ventricular ejection fraction; GLS, global longitudinal strain. *Significant difference (*p *< 0.05) *vs.* baseline.

### 3.3 LV Characteristic Changes after Surgery

Postoperative LVEF ranged between 20% and 64%. Changes in LV size (LVEDD, LVESD) and LVEF evaluated by TTE are shown in Table 3. The postoperative LVEDD, LVESD and LVEF were significantly improved from the preoperative data (62.6 ± 12.3 mm *vs.* 76.2 ± 8.2 mm for LVEDD, *p *< 0.001; 49.7 ± 15.1 mm *vs.* 65.1 ± 7.7 mm for LVESD, *p *< 0.001; 42.4 ± 13.3% *vs.* 26.2 ± 5.3% for LVEF, *p *< 0.001, respectively), while the LV was still enlarged and LVEF was still decreased.

**Table 3.** **Changes in echocardiographic characteristics after AVR (n = 37)**.

Variables	Pre-surgery	Post-surgery	*p* value
LVEDD (mm)	76.2 ± 8.2	62.6 ± 12.3	<0.001
LVESD (mm)	65.1 ± 7.7	49.7 ± 15.1	<0.001
LVEF (%)	26.2 ± 5.3	42.4 ± 13.3	<0.001

Data are expressed as mean ± SD. LVEDD, left ventricular end-diastolic diameter; LVESD, left ventricular end-systolic diameter; LVEF, left ventricular ejection fraction.

### 3.4 Comparison of Variables in the Well-Recovery and Poor-Recovery Groups

All post-surgery cases were divided into a well-recovery group (post LVEF >40%, n = 18) and a poor-recovery group (post LVEF ≤40%, n = 19) based on LVEF derived from TTEs. In terms of clinical data and prosthetic valve type, there were no statistical differences between the two groups. During DSE, for conventional echocardiographic data, baseline LVEDD, baseline LVESD, baseline LVEF, peak LVEF and ΔLVEF in the well-recovery group were better than those of the poor-recovery group, but only baseline LVESD was statistically different. For STE data, the well-recovery group had higher baseline GLS and peak GLS than the poor-recovery group (–8.6 ± 1.6% *vs.* –6.5 ± 1.2% for baseline GLS, *p *< 0.001; –10.6 ± 1.7% *vs.* –8.2 ± 1.2% for peak GLS, *p *< 0.001, respectively), but ΔGLS between the two groups were similar with no statistical differences (Table 4).

**Table 4.** **Pre-surgery and follow-up Characteristics between well-recovery and poor-recovery groups**.

Variables		Well-recovery group (n = 18)	Poor-recovery group (n = 19)	*p* value
Clinical data				
	Age (yrs)	53 ± 9	57 ± 10	0.262
	Male, n (%)	15 (83)	17 (89)	0.585
	Hypertension, n (%)	9 (50)	5 (26)	0.138
	Diabetes, n (%)	2 (11)	3 (16)	0.677
	NYHA class (≥II), n (%)	16 (89)	15 (79)	0.412
	Heart rate (bpm)	74 ± 13	77 ± 11	0.405
	AF, n (%) *	1 (6)	3 (16)	0.604
Valve type *				
	Mechanical valve, n (%)	15 (83)	13 (68)	0.291
	Biological valve, n (%)	3 (17)	6 (32)	
Echocardiographic data				
	Baseline LVEDD (mm)	74.1 ± 8.0	78.2 ± 8.0	0.139
	Baseline LVESD (mm)	61.7 ± 6.0	68.3 ± 7.9	0.008
	Baseline LVEF (%)	27.6 ± 5.7	24.8 ± 4.6	0.122
	Peak LVEF (%)	38.0 ± 5.5	34.3 ± 6.3	0.070
	ΔLVEF (%)	10.4 ± 2.7	9.5 ± 2.9	0.318
	Vmax (m/s) *	2.5 ± 0.34	2.6 ± 0.38	0.563
	Maximum PG (mmHg) *	26.7 ± 7.6	27.9 ± 8.4	0.677
	Mean PG (mmHg) *	14.8 ± 4.2	16.1 ± 5.0	0.410
STE data				
	Baseline GLS (%)	–8.6 ± 1.6	–6.5 ± 1.2	<0.001
	Peak GLS (%)	–10.6 ± 1.7	–8.2 ± 1.2	<0.001
	ΔGLS (%)	–1.9 ± 1.2	–1.8 ± 0.9	0.703

Data are expressed as mean ± SD or as n (%). NYHA, New York Heart Association; AF, atrial fibrillation; LVEDD, left ventricular end-diastolic diameter; LVESD, left ventricular end-systolic diameter; LVEF, left ventricular ejection fraction; Vmax, maximum transvalvular velocity for prosthetic aortic valve; PG, pressure gradient for prosthetic aortic valve; STE, speckle tracking echocardiographic; GLS, global longitudinal strain. * These data were postoperative results.

### 3.5 Predictors of Postoperative LV Systolic Function

In simple linear regression analysis, baseline GLS and peak GLS correlated better with post-surgery LVEF (R = –0.581 for baseline GLS, *p *< 0.001; R = –0.596 for peak GLS, *p *< 0.001; respectively) than did baseline LVESD and baseline LVEDD (R = –0.543 for baseline LVESD, *p *<0.001; R = –0.355 for baseline LVEDD, *p* = 0.031) (Table 5). Among baseline LVEDD, baseline LVESD, baseline GLS, peak GLS, age and gender, logistic regression analysis using stepwise algorithm demonstrated that baseline GLS and peak GLS were independent predictors of marked recovery of LVEF among the covariates examined (*p* = 0.049 for baseline GLS and 0.020 for peak GLS, respectively; $R^{2}$ = 0.640).

**Table 5.** ** Univariate analyses between pre-surgery variables and post-surgery LVEF**.

Pre-surgery variables		Correlation coefficient	*p* value
Echocardiographic data			
	Baseline LVEDD (mm)	–0.355	0.031
	Baseline LVESD (mm)	–0.543	<0.001
	Baseline LVEF (%)	0.219	0.192
	Peak LVEF (%)	0.296	0.075
	ΔLVEF (%)	0.238	0.156
STE data			
	Baseline GLS (%)	–0.581	<0.001
	Peak GLS (%)	–0.596	<0.001
	ΔGLS (%)	–0.059	0.728

LVEDD, left ventricular end-diastolic diameter; LVESD, left ventricular end-systolic diameter; LVEF, left ventricular ejection fraction; STE, Speckle tracking Echocardiography; GLS, global longitudinal strain.

**Fig. 4.**
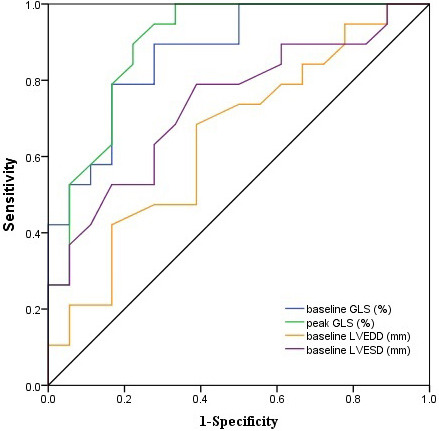
** The ROC curves for predicting marked recovery of LV contractive function (follow-up LVEF >40%)**. Baseline GLS showed AUC = 0.868, sensitivity and specificity were 89.5% and 72.2%, respectively. Peak GLS showed the highest AUC = 0.895, sensitivity and specificity were 89.5% and 77.8%, respectively. ROC, receiver-operating characteristic; LV, left ventricular; LVEF, left ventricular ejection fraction; GLS, global longitudinal strain; AUC, area under the curve; LVEDD, left ventricular end-diastolic diameter; LVESD, left ventricular end-systolic diameter.

The prediction performance of conventional echocardiographic parameters and STE parameters for marked recovery of LV contractive function (follow-up LVEF >40%) was determined by ROC curves (Table 6, Fig. 4). The area under the curve (AUC) of STE parameters were significantly larger than that of conventional echocardiographic parameters. Baseline GLS showed a strong predictive value (AUC = 0.868), the cutoff value was –7.8%, and the corresponding sensitivity and specificity were 89.5% and 72.2%, respectively. Furthermore, peak GLS showed the highest predictive value (AUC = 0.895), the cutoff value was –9.4%, and the corresponding sensitivity and specificity were 89.5% and 77.8%, respectively.

**Table 6.** **ROC analyses for prediction of marked recovery of LVEF by pre-surgery parameters**.

Variables	AUC (95% CI)	Cutoff value	Sensitivity, %	Specificity, %	*p* value
Baseline LVEDD (mm)	0.649 (0.471–0.828)	75.5	68.4	61.1	0.121
Baseline LVESD (mm)	0.738 (0.577–0.899)	64.5	63.2	72.2	0.013
Baseline GLS (%)	0.868 (0.755–0.982)	–7.8	89.5	72.2	<0.001
Peak GLS (%)	0.895 (0.789–1.000)	–9.4	89.5	77.8	<0.001

Data are expressed as mean ± SD. AUC, area under the curve; LVEDD, left ventricular end-diastolic diameter; LVESD, left ventricular end-systolic diameter; GLS, global longitudinal strain.

## 4. Discussion

AR occurs secondary to primary aortic valve lesions or geometric changes in the aortic root, commonly in degenerative diseases, rheumatic heart disease, and congenital abnormalities [19]. Chronic severe AR causes excessive LV volume overload and end-diastolic pressure which can lead to LV enlargement and LV contractile dysfunction [20]. According to the guidelines, it is necessary for patients with chronic severe AR and significantly decreased LVEF to undergo surgical AVR [16, 21]. This specific patient population has a higher perioperative mortality than those with normal or mild-moderately reduced LVEF. The short-term recovery of LV contractile function after AVR is closely related to long-term prognosis [3, 5, 22]. Therefore, it is important to be able to accurately predict the short-term recovery after AVR in patients with reduced LVEF to determine which patients will derive the greatest benefit from surgery.

Stress echocardiography (SE) is a commonly used, non-invasive, convenient and reliable method for evaluating LV CR in clinical practice. Several studies have shown that in patients with severe AR, LV CR based on conventional echocardiographic parameters could predict the recovery of LV contractile function after surgery [12, 22, 23]. In our study, all surgical patients were assessed with LV CR based on conventional echocardiographic parameters for LVEF. During short-term follow-up, LVEF and LV size markedly improved compared with pre-surgery data in the entire group. This reverse remodeling of LV demonstrated that some patients could benefit from surgery. Moreover, only one patient experienced a perioperative death because of respiratory failure. This low perioperative mortality may be related to our strict definition of LV CR (ΔLVEF ≥8%), contemporary improvement in surgical techniques and high-quality perioperative management.

However, we also found that the recovery of LVEF varied significantly and only half of these patients improved to an LVEF >40%. In this study, LV CR based on LVEF could not accurately predict marked recovery of LV contractile function after AVR, which was not completely consistent with previous studies. This could be explained by the difference between the current study population and previous ones, who had normal or mild-moderately reduced LVEF and smaller LV size before surgery. In the significantly enlarged LV due to chronic overload, LVEF cannot accurately reflect LV contractile function and reserve, leading to difficulty in predicting marked recovery after AVR. This is because the evaluation of LVEF was based on the change of the chamber volume, which reflected the geometric change and the overall LV contractile function rather than the intrinsic contractile function of the myocardium [24, 25, 26].

Conventional echocardiographic parameters reflect the functional structure of the left ventricle as a whole, but cannot reflect the function of the local myocardium. The contraction of the LV myocardium involves multiple directions including longitudinal, radial, circumferential, and torsional one. They act simultaneously to constitute the overall contractile activity of the LV. STE can quantitatively analyze the myocardial strain in a specific direction, such as GLS, which is in the longitudinal direction [27]. Therefore, STE is currently a widely used tool for evaluating intrinsic contractility of the myocardium as it can track the movement of the myocardium and detect subtle changes at the myocardial level [27, 28]. Previous studies have shown that myocardial strain based on STE is more sensitive in evaluating LV systolic dysfunction in patients with chronic AR than volume-based LVEF [14, 29, 30, 31]. In addition, due to the high reproducibility and feasibility of GLS, it has been suggested as a diagnostic tool to evaluate LV CR [10, 32].

In our study, both the baseline GLS and peak GLS during low-dose DSE were significantly lower than the normal value (<–20% [32]), suggesting severe impairment of LV systolic function in these patients. Alashi *et al*. [27] and Olsen* et al*. [31] demonstrated impaired GLS was strongly associated with prognosis in patients with severe AR. In addition, we found both baseline GLS and peak GLS of the well-recovery group were better than those in the poor-recovery group. This difference demonstrates that myocardial strain analysis is consistently more sensitive to detect myocardial damage than LVEF in the cases of severe AR with significant LV contractile dysfunction.

Both baseline GLS and peak GLS showed higher predictive value than conventional echocardiographic indices for predicting postoperative recovery in these patients. Furthermore, peak GLS had the highest predictive ability (AUC: 0.895; sensitivity: 89.5%; and specificity: 77.8%). This may be due to the fact that peak GLS reflects both the baseline and reserved contractility of the LV, which is revealed by the combination of DSE and STE. The preoperative peak GLS may better determine the level of recovery of LVEF after AVR in patients with chronic severe AR and severe LV contractile dysfunction.

Myocardial deformation could be assessed by speckle tracking technologies including 2D and 3D STE. 2D-STE has been shown to be able to effectively detect subtle systolic function impairment in a variety of diseases [33, 34]. One previous study [35] found that in asymptomatic chronic AR patients with preserved LVEF, strain parameters acquired by 3D-STE were basically consistent with 2D-STE and feature tracking magnetic resonance imaging. This confirms that 3D-STE is highly reliable in such patients. In addition, 3D-STE allows the quantification of complex ventricular mechanics including torsion, twist and area strain, which could not be reliably assessed by 2D-STE. 3D-STE is also free from the influence of out-of-plane motion in 2D echocardiography. However, 3D-STE is subject to technical limitations including very low temporal and spatial resolution, intervendor differences and non-standardization [36]. Future clinical studies investigating the added prognostic value of 3D-STE in the current patient population are promised.

## 5. Limitations

It should be noted that this study has some limitations. First, due to the strict enrollment criteria of this study in a single center, the sample size was relatively small. With this relatively low number of patients involved, only limited consequences could come out. Thus, this study could be considered as a preliminary validation of the feasibility of STE combined with low-dose DSE in predicting the surgical outcome in patients with chronic severe AR and markedly reduced LV function. Future studies with larger sample size and more definite outcome events are guaranteed. Second, this is a trial with retrospective design and is thus subject to its innate limitations. Prospective studies are needed to verify the current findings. Third, TTE and STE were all 2D-based in this study. The influence of out-of-plane motion was especially prominent in significantly enlarged LV and may prevent accurate assessment of strain parameters. 3D TTE and STE is not subject to such influence and could be considered a promising research direction.

## 6. Conclusions

Patients with chronic severe AR and markedly reduced LV function who demonstrate LV CR could benefit from surgical AVR. STE combined with DSE could provide a more sensitive quantitative index for predicting the recovery of LV systolic function after AVR in this patient population. Due to the non-invasive, convenient and accurate characteristics of this combined method, it would be expected to become a new means for clinical application to evaluate LV contractile function and CR, and may be an important reference for clinical decision-making.

## Data Availability

The datasets used and/or analyzed during the current study are available from the corresponding author on reasonable request.
